# Association between food insecurity and overweight: Protocol for a systematic review based on intersectionality of gender and race/color

**DOI:** 10.1371/journal.pone.0315184

**Published:** 2024-12-26

**Authors:** Renatha Celiana da Silva Brito, Poliana de Araújo Palmeira, Jackson Silva Lima Laurentino, Rônisson Thomas de Oliveira Silva, Ana Beatriz Macêdo Venâncio dos Santos, Angelo Giuseppe Roncalli da Costa Oliveira

**Affiliations:** 1 Doctoral Candidate in the Graduate Program in Public Health, University of Rio Grande do Norte (UFRN), Natal, Rio Grande do Norte, Brazil; 2 Federal University of Campina Grande (CES/UFCG), Cuité, Paraíba, Brazil; 3 Doctoral Candidate in the Graduate Program in Nutrition Sciences, Federal University of Paraíba (UFPB), João Pessoa, Paraíba, Brazil; 4 PhD in the Postgraduate Program in Public Health, University of Rio Grande do Norte (UFRN), Natal, Rio Grande do Norte, Brazil; 5 Federal University of Rio Grande do Norte (UFRN), Natal, Rio Grande do Norte, Brazil; School of Nursing Sao Joao de Deus, Evora University, PORTUGAL

## Abstract

Food insecurity violates the right to regular access to quality food, affecting population groups unequally. In Brazil, FI is associated with both malnutrition and increased obesity and is intertwined with racial and gender inequalities, perpetuating cycles of poverty and social exclusion. This protocol aims to select observational studies that evaluate the association between food insecurity and overweight and their intersectional discussions (gender and race/color). The PRISMA checklist guidelines will be applied, and the PROSPERO platform will be used for registration. Searched in the Virtual Health Library (Lilacs-BVS), Medline PubMed, Web of Science, and Embase Elsevier databases. The stages of article selection and information extraction will be carried out by independent researchers who will identify articles that meet the established inclusion criteria, removing duplicate publications and excluding those that do not meet the requirements. The quality of eligible articles will be assessed using the Quality Assessment Tool For Quantitative Studies (QATFQS), which is recommended for observational studies. This study is not needed for ethical approval, as it is a systematic review based on secondary data. They will disseminate their conclusions from the original articles.

**PROSPERO registration number:** CRD42023427239.

## Introduction

The concept of Food Security and Nutrition (FSN) is related to the right of every individual to regular and permanent access to healthy food from a qualitative and quantitative point of view (food of good quality and in sufficient quantity to meet basic nutritional needs) [1] so that this access does not compromise other essential needs, such as housing, health, education, sanitation, and transportation [2].

Therefore, Food Insecurity (FI) is defined as an economic and social condition covering households with limited or uncertain access to healthy food [1], with hunger being the individual-level physiological condition that can result from this FI [3]. It is a social and historical problem affecting millions worldwide. According to the Food and Agriculture Organization (FAO), in 2022, around 2.4 billion people did not have secure and permanent access to food, with an increased impact on their family incomes due to the COVID-19 pandemic. This number is relatively higher for women and rural communities [4]. In Latin America and the Caribbean, hunger affected around 47.7 million people in 2019. It made health organizations warn about the significant increase in FI in this region, which grew progressively between 2014 (22.9%) and 2022 (37.5%) for moderate and severe FI, with a sharp increase in South America [4, 5].

FI is associated with different social vulnerabilities, such as gender inequality, unemployment, poverty, low schooling, limited access to health services, climate change, lack of basic sanitation, social discrimination, racism, restricted access to land, technologies, and agricultural innovations [6–8]. In addition, it is also associated with health conditions, such as malnutrition, nutritional deficiencies, and Chronic Non-Communicable Diseases (CNCD), among which obesity stands out [5, 9]. According to the *Vigilância de Fatores de Risco e Proteção para Doenças Crônicas por Inquérito Telefônico* (VIGITEL) [10], in 2023, Brazil had 24.3% of adults Brazilians with obesity, higher in women.

The Brazilian and international literature has recently discussed the association between FI and being overweight. This association is related, in particular, to poor nutrition, from the high consumption of processed and ultra-processed foods, which are poor in nutrients, high in energy density, and rich in sugars, salt, and saturated fats, as well as issues such as sedentary lifestyles and chronic stress [11, 12]. When it comes to the association between race and overweight, although this relationship has yet to be fully elucidated [13], some studies demonstrated a statistically significant association between overweight and self-reported race/color in adult women from the Northeast regions [14] and Central-West of Brazil [15].

By definition, color refers to observable phenotypic characteristics, such as skin tone, hair, and eyes [16], and these classifications can vary significantly between countries as they reflect distinct historical, social, and cultural contexts. In Brazil, the classification of race and color demonstrates a complex interaction between social identity, self-identification, and ancestry [17].

Race, on the other hand, is a more complex concept that involves a social construction [16]. Although biological characteristics initially defined it, it is currently understood as a sociological category reflecting common ancestry and societal power relations. Race describes social groups that share a collective history and face racial inequalities [18]. Thus, in Brazil, national research institutes and the government have adopted self-declaration of race and skin color as a way of capturing the racial and ethnic diversity of the population and monitoring social and economic inequalities between different racial groups [19].

Regarding the relationship between “gender,” “race and color,” and “FI” in Brazil, the rates of moderate and severe FI were higher in households headed by black women, according to the II National Survey on Food Insecurity in the Context of the COVID-19 Pandemic in Brazil (II VIGISAN) [20]. This perspective is based on the matrices of domination, that is, on the complex power structures that involve the various forms of oppression and the recognition of resistance [21], understanding the paths that lead black women, for example, to face specific challenges about FI [22].

Thus, when analyzing the intersections of race and color in the Brazilian context, it is essential to recognize the overlap of these identities in social and health disparities. In this sense, there is an urgent need to study the long-term implications of these overlaps in FI. Gender and race/color disparities perpetuate cycles of poverty and inequality and negatively affect future generations, exposing them to more significant risk factors, malnutrition, and overweight. Studying the associations between FI and overweight, in this case, from the perspective of gender and race/color issues, will help public policies tackle FI in a more comprehensive and effective way.

The guiding question of the present study is: "What is the state of knowledge about the intersectionality of gender and race/color in the association of FI with excess weight in the Brazilian population of men and women over 18 years of age?" The objective of this protocol, and of the following systematic review, is to analyze the association between FI and overweight under the discussion of intersectionality in the Brazilian population, that a majority of black and brown people (55.5%) and women (51.5%) [23]. Although this discussion is already a little more advanced internationally [24–29], there is a gap in studies on this subject in Brazil.

## Methods

### Study registration

This protocol follows the PRISMA (Preferred Reporting Items for Systematic Reviews and Meta-Analysis) checklist guidelines [30, 31], and the systematic review was registered on the PROSPERO (International Prospective Register of Systematic Reviews) platform on May 27, 2023, under protocol CRD42023427239. Available at: https://www.crd.york.ac.uk/prospero/display_record.php?ID=CRD42023427239.

### Electronic searches

The bibliographic search was carried out in the Biblioteca Virtual de Saúde (BVS), PubMed, Web of Science, and Embase databases to find observational studies evaluating the association between FI and overweight. A reverse citation search will also be carried out, in which other articles not identified in the initial search of the databases will be included manually from the list of references and citations of the articles.

### Inclusion and exclusion criteria

The following inclusion criteria will be considered: (1) studies conducted with adults and elderly individuals from the Brazilian population (aged 18 or over) of both sexes (men and women); (2) that used the Brazilian Food Insecurity Scale (EBIA) to measure the prevalence of FI, either as a stage or exposure variable, and regardless of whether it was categorical or docytomic; (3) that used an excess weight variable, either as a stage or exposure variable and regardless of whether it was categorical or docytomic; (4) that analyzed the association (by association tests) between FI and excess weight (stratified by sex); (5) that used validated measures to classify nutritional status; (6) and observational studies (cross-sectional, longitudinal).

Regarding the variables of gender, race, and color, these will not be inclusion criteria, given that the objective is precisely to identify whether or not the studies that evaluated the association between FI and excess weight included these factors in the analyses, either as a determining variable or simply as a methodological adjustment adding to other confounding factors, such as education and socioeconomic conditions.

In addition, when using or not using these intersectional factors in your studies, try to observe how this discussion has progressed in each of them. In this sense, articles that meet the eligibility criteria based on Population, Exposure, Comparison, Outcome, and Study Design (PECO), described in Table 1, will be included.

**Table 1:** PECO description for this systematic review, Brazil, 2024.

Abbreviation	PECO	Elements
P	Participants	Brazilian men and women, adults and seniors (≥ 18 years old).
E	Exposure	Food insecurity will be analyzed using the EBIA (8 or 14 questions), which classifies the degree of FI as mild, moderate, or severe. Overweight will be classified using validated measures for classifying nutritional status.
C	Comparison	Exposure will be compared between “white men”, “black and brown men”, “white women” and “black and brown women”.
O	Outcome	How the intersectionality of gender and race/color permeate the association between FI and overweight.

Source: Elaborated by the authors, 2024.

Qualitative, ecological, case-control studies, literature reviews, preprint articles, validation studies, and analyses of EBIA cut-off points, theses, and dissertations in repositories were excluded. Studies with pregnant women, children, adolescents, and hospitalized individuals or those suffering from contagious and infectious diseases were also excluded.

### Search strategy

The terms used are descriptors indexed in the DeCS/MeSH system and natural language keywords. All were researched and selected based on their relevance in national and international literature to ensure that as many studies as possible align with the theme of the proposal. The terms and their respective IDs were as follows: “Obesity” (D009765), “Overweight” (D050177), “Overnutrition” (D044343), “Nutritional Status” (D009752), “Body Mass Index” (D015992), “Food Insecurity” (D000084884), “Food Security” (D000082302), “Food Supply” (D005523), “Brazil” (D001938), and the keywords “Household Food Insecurity” and “Household Food Security”.

The final search was as described below: “Obesity” (DeCS/MeSH Terms); OR “Overweight” (DeCS/MeSH Terms); OR “Overnutrition” (DeCS/MeSH Terms); OR “Body Mass Index” (DeCS/MeSH Terms); AND “Food Insecurity” (DeCS/MeSH Terms); OR “Food Security” (DeCS/MeSH Terms); “Food Supply” (DeCS/MeSH Terms); OR “Household Food Security” (Title/Abstract); OR “Household Food Insecurity” (Title/Abstract); AND “Brazil” (DeCS/MeSH Terms).

Although the aim is also to discuss the intersectionality of the association between FI and overweight, there was a significant limitation to including the terms related to intersectionality in the search strategy. Intersectionality is a term proposed by African-American Kimberlé W. Crenshaw to designate the interdependence of race, gender, and class power relations, and currently described as a transdisciplinary theory that aims to understand the complexity of identities and social inequalities [32].

The limitations found are related to its broad concept and subjectivity in the literature, the recent inclusion of this topic in scientific discussions, its scientific gap, and the impossibility of understanding the experiences of oppression and discrimination in isolation but instead analyzed in terms of complex interactions.

The keywords related to intersectionality were not included in the search strategy, given the restrictions and limitations attributed to its results—"class", "race", "gender", "sexism", "racism", "intersectional", "female", "women", "color", "species". And the decision was made to leave them reserved for the essential reference for the discussion, which will undoubtedly expand the study of these issues for public health, and generate possibilities.

### Study selection process and extraction

Once the articles have been identified by searching the databases and based on the inclusion criteria, the following steps will be carried out: (1) Removal of duplicate articles; (2) Screening of the articles by reading the titles and abstracts; (3) Reverse search, in which other articles not identified in the initial search of the databases will be included manually from the list of references and citations of the articles; (4) Full reading of the articles screened; (5) Definition of the articles eligible for quality analysis (6) Quality analysis using the Quality Assessment Tool For Quantitative Studies (QATFQS) instrument [33] recommended for observational studies; (7) Definition of the articles included in the review.

**Fig 1:**
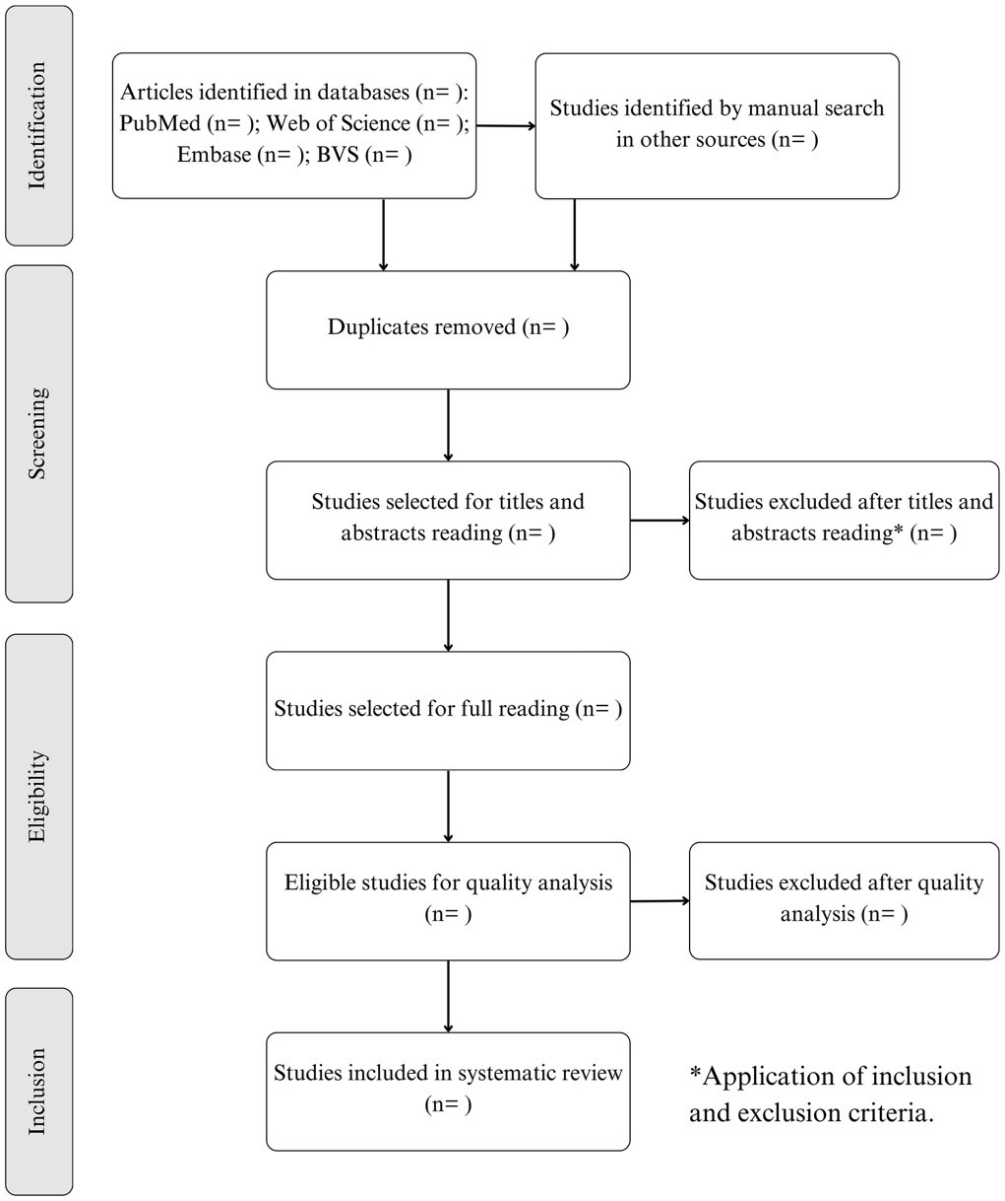
Flowchart of the selection process for the articles included in this systematic review, Brazil, 2024. Source: Elaborated by the authors, adapted from PRISMA, 2024.

The ENDNOTE software, the online version, will be used to remove duplicate articles and organize the references. The selection and quality analysis of the articles will be carried out by two independent researchers (RCdSB and RTdOS); in the event of disagreement between them, even after a consensus discussion, a third researcher will be consulted. Fig 1 shows the flowchart adapted from PRISMA containing all the stages of article selection for this review.

Data extraction will also be carried out by two independent researchers (JSLL and ABMVdS), and the information to be extracted will follow a protocol previously established by the authors, which will guide the reading of the article to identify indicators used to assess FI, nutritional status and their relationship with gender/sex and race/color.

The methodological and results information from the manuscripts to be extracted and systematized will be: authors; year of publication; study objective; data source (primary, secondary data); type of study (cross-sectional, longitudinal), and sample composition (probabilistic, non-probabilistic sample); study population and year of data collection; study location (city/state and area of residence); measure of nutritional status (Body Mass Index—BMI, other); statistical analyses carried out and control/adjustment variables; main results on the association between FI and overweight (and whether these were associated with the variables of sex, color/race); hypotheses or justifications for the association between FI and overweight (and discussion of intersectionality, if any).

### Methodological quality assessment

The QATFQS [33], recommended for observational studies, will be used to assess the quality of evidence from eligible articles, considering five components: a) selection bias; b) study design; c) confounders; d) data collection methods; e) withdrawals.

For each component, around two to three questions must be answered according to the level of adequacy in the article’s provisions. At the end of each element, an assessment is made as to whether that particular session was considered "strong", "moderate", "weak" in its adequacy, or "not applicable" (if the article does not include information on that session).

Finally, a table is filled in which will classify the quality of the articles as "strong," "moderate," and "weak" in terms of evidence related to the sample, inconsistencies, and accuracy of results, among other factors. Articles with a "weak" rating will not be included.

### Data synthesis

Agreement between the researchers will be assessed using the kappa index [34], with the agreement being determined when the two researchers choose to exclude or include the same article. As this is not a meta-analysis, other statistical analyses will not be conducted using the included articles.

The data generated from the results of this systematic review will be used in the manuscripts to be published in scientific journals. The information in this protocol is unpublished, and to date, no results have been published or submitted on the outcome of this research.

## Discussion

In Brazil, the EBIA measures and classifies the FI as mild, moderate, and severe [35]. FI is a household-level economic and social condition of limited or uncertain access to adequate food and a potential risk factor for malnutrition and, paradoxically, obesity in Brazil and worldwide [11, 26, 29]. The BMI is one way to measure nutritional status through the ratio between body weight and height squared, which classifies an individual’s nutritional status as underweight, eutrophic, overweight, and severely obese [36].

According to Mazur and Navarro [37], in developing countries such as many in Latin America, finding strategies to tackle the problem of the relationship between FI and obesity is a challenge for governments and health professionals, because individuals with an anthropometric state of obesity also have nutritional deficiencies and high rates of malnutrition, i.e., in a situation of double burden of malnutrition [9, 38].

Intersectionality, as overlaps of gender and race/color, permeates structural inequalities, as these factors interact with other social, economic, and cultural dimensions and create systems of oppression and inequality, as well as unequal access to resources. The intersectionality highlights how women and people from different racial backgrounds have unequal access to education, employment, fair wages, and health services, which affect their ability to acquire adequate and nutritious food for themselves and their families.

Regarding the racial classification used in the studies, since the object of investigation is Brazilian research, it is believed that this variable is included chiefly based on the scale defined and recommended by the IBGE, which, since 1991, the Brazilian Institute of Geography and Statistics (IBGE) has adopted five main categories to classify color or race (white, black, brown, Asian, and indigenous), allowing individuals to self-identify, that is, to choose how they perceive themselves racially or based on their skin color. This implementation of the “race/color” item by self-declaration has provided that the identification of the black and brown population has increased significantly in the last three decades [19, 39, 40]. This differs from countries such as the United States, which uses the following classification: white, black or African-American, Native American, Asian, Hispanic, or Latino (considered an ethnicity, not a race) [41, 42].

In 2022, Brazil completed 134 years since the end of the period of slavery in the country, which lasted for 388 years [43]. However, it was only at the end of the 20th century that race began to be considered a social determinant of health among studies related to health issues, highlighting disparities in access and progression of diseases [44]. From this, there is now a large body of literature that highlights racism as a factor that drives racial inequalities in the area of health.

The dimensions of race, color, and ethnicity are fundamental to understanding how health outcomes are distributed among the population and, thus, to inform public policies [45]. The inequalities of class, gender, and race/color that affect FI and excess weight are directly related to structural racism and historical disparities in Brazil. This reveals the overlapping factors that impact the unequal distribution of resources and opportunities for permanent access to health and food, exposing groups more vulnerable to higher rates of discrimination and FI [17].

Following the principles of health equity, the National Policy for Comprehensive Health of the Black Population was established by the Ministry of Health through Ordinance No. 992/2009 to promote the comprehensive health of the black population and prioritize the reduction of ethnic-racial inequalities and the fight against racism and discrimination, recommending the inclusion of the “race/color” item in all data collection instruments of public services and suggesting the analysis of disaggregated data, through Ordinance No. 344/2017 [46–48]. In addition, these data can be used in different ways (dichotomous or categorical), depending on the objective to be analyzed and the sample [17].

Intersectionality theory provides a valuable framework for understanding how different social identities, including race and color, overlap and interact in social and health disparities. This approach justifies the use of the terms “race” or “race/color” rather than just “color” or “ethnicity,” as the latter are not fully capable of capturing and understanding aspects of: i) the complexity of identities, in the recognition that identity is not something fixed and determined, but rather a social and historical construction, for example the category “pardo,” which is related to individuals with dark or mixed skin, often described as “mestizos,” and includes those with both African and indigenous ancestry, reflecting the racial diversity of the Brazilian population; ii) the power relations that shape the experiences of different groups, since the term “race” implies the recognition of the historical structures of oppression that affect racialized groups, and how this perspective manifests itself in areas such as food security; iii) self-identification and recognition, since the use of the terms “race” or “race/color” allows individuals to identify themselves in a way that resonates with their personal and collective experiences, being crucial for the collection of accurate statistical data that formulate public policies [17, 18, 44, 45, 49].

When studying the impact that self-reported race and race collected by the interviewer can have on income inequality in the United States population, Sapertein [50] observed that, even though the agreement between self- and heteroidentified race information is over 96%, there are still differences when analyzing the degree of inequality based on the two classifications, which can lead to different conclusions. In contrast, in the Brazilian population, studies have already observed that ethnic-racial inequalities are slightly higher when the classification is made by hetero-identification [51, 52].

Historically marginalized groups, such as black and brown people, especially women, face a double burden of discrimination, both because of their gender and their skin color, which amplifies the effects of racist policies and socioeconomic inequality. Intersectionality, such as overlapping gender and race/color, permeates structural inequalities since these factors interact with other social, economic, and cultural dimensions and create systems of oppression and inequality, as well as unequal access to resources.

In this sense, the potential impact of the results of this review is directed toward understanding the relationship between excess weight and food insecurity in the Brazilian population, as well as other related factors that can be identified (e.g., education, family composition, age, occupation, access to government benefits and income transfer programs, and access to health care).

By identifying these aspects in Brazil, especially in areas of social vulnerability, it becomes possible to understand and discuss the existing weaknesses in the implementation of public policies related to access to health care, food security, and income, expanding the scope of existing policies and encouraging equitable policies for vulnerable groups.

## Supporting information

S1 Checklist PRISMA 2015 checklist recommended items to address in a systematic review protocol. (DOCX)
